# Crystal structure of bis­[μ-*S*-hexyl 3-(2-oxido­benzyl­idene)di­thio­carbazato-κ^4^ *O*,*N* ^3^,*S*:*O*]dicopper(II)

**DOI:** 10.1107/S2056989015022914

**Published:** 2015-12-09

**Authors:** M. S. Begum, M. B. H. Howlader, M. C. Sheikh, R. Miyatake, E. Zangrando

**Affiliations:** a Department of Chemistry, Rajshahi University, Rajshahi-6205, Bangladesh; b Department of Applied Chemistry, Faculty of Engineering, University of Toyama, 3190 Gofuku, Toyama 930-8555, Japan; c Center for Environmental Conservation and Research Safety, University of Toyama, 3190 Gofuku, Toyama 930-8555, Japan; d Department of Chemical and Pharmaceutical Sciences, via Giorgieri 1, 34127, Trieste, Italy

**Keywords:** crystal structure, Schiff base, binuclear copper(II) complex, di­thio­carbazate ligand

## Abstract

The title compound, [Cu_2_(C_14_H_18_N_2_OS_2_)_2_], is a binuclear copper(II) complex of an oxybenzyl­idenedi­thio­carbazate ligand. The ligand coordinates in a tridentate manner through N-, S- and O-donor atoms. Each O atom also bridges to a second Cu^II^ ion to form the binuclear species. It has a central Cu_2_O_2_ rhomboid moiety and a metal-to-metal separation of 2.9923 (6) Å. In the crystal, the binuclear complexes stack along the *a* axis with all the hexyl chains located side-by-side, forming a hydro­phobic region. The complexes are linked *via* C—H⋯N hydrogen bonds, forming chains along the *c*-axis direction. One Cu^II^ atom has the S atom of a symmetry-related complex located approximately in the apical position at 2.9740 (11) Å. This weak inter­action links the chains to form slabs parallel to the *ac* plane.

## Related literature   

For details of the bioactivities of metal complexes of bidentate Schiff bases of *S*-methyl or *S*-benzyl di­thio­carbazate ligands, see: Chan *et al.* (2008 ▸); How *et al.* (2008 ▸); Ali *et al.* (2002 ▸); Chew *et al.* (2004 ▸). For square-planar metal complexes of di­thio­carbazate ligands coordinating in a bidentate manner, see: Tarafder *et al.* (2008 ▸); Howlader *et al.* (2015 ▸); Begum *et al.* (2015 ▸). For Cu—N and Cu—S bond lengths in mononuclear bis-chelated species, see: Zangrando, Begum *et al.* (2015 ▸); Zangrando, Islam *et al.* (2015 ▸). For copper(II) complexes of similar ligands, see: Ali, Tan *et al.* (2012 ▸); Ali, Mirza *et al.* (2012 ▸).
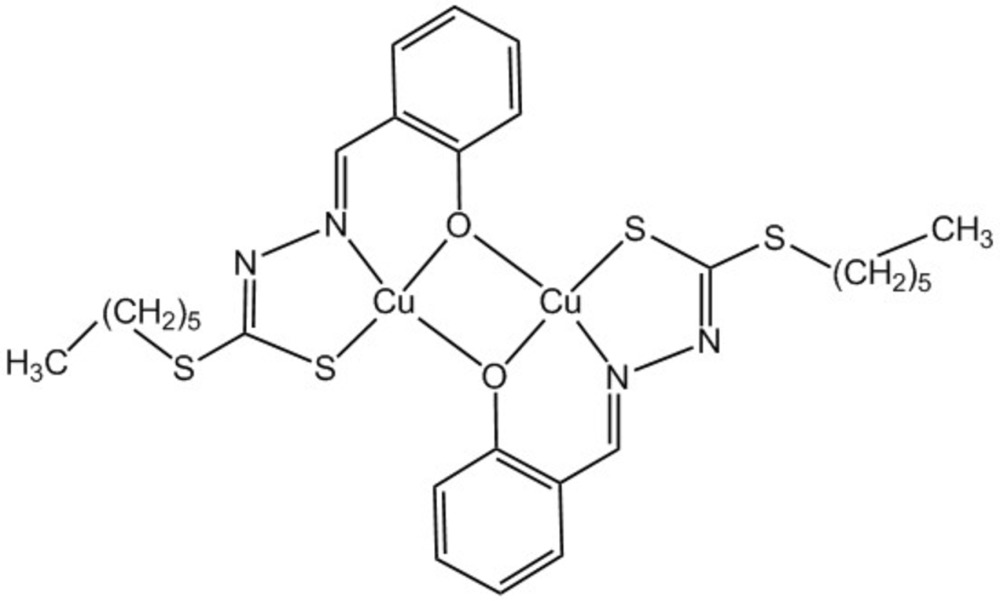


## Experimental   

### Crystal data  

[Cu_2_(C_14_H_18_N_2_OS_2_)_2_]
*M* *_r_* = 715.93
Monoclinic, 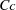

*a* = 7.2792 (4) Å
*b* = 37.7252 (16) Å
*c* = 11.3443 (5) Å
β = 94.701 (2)°
*V* = 3104.8 (3) Å^3^
*Z* = 4
Mo *K*α radiation
μ = 1.67 mm^−1^
*T* = 173 K
0.36 × 0.34 × 0.03 mm

### Data collection  

Rigaku R-AXIS RAPID diffractometer
Absorption correction: multi-scan (*ABSCOR*; Higashi, 1995 ▸) *T*_min_ = 0.723, *T*_max_ = 0.951
12702 measured reflections
5278 independent reflections
5114 reflections with *I* > 2σ(*I*)
*R*_int_ = 0.025

### Refinement  

*R*[*F* ^2^ > 2σ(*F* ^2^)] = 0.027
*wR*(*F* ^2^) = 0.066
*S* = 1.06
5278 reflections
361 parameters
2 restraints
H-atom parameters constrained
Δρ_max_ = 0.60 e Å^−3^
Δρ_min_ = −0.29 e Å^−3^
Absolute structure: Flack *x* determined using 2223 quotients [(*I* ^+^)−(*I* ^−^)]/[(*I* ^+^)+(*I* ^−^)] (Parsons *et al.*, 2013 ▸)
Absolute structure parameter: 0.006 (6)

### 

Data collection: *RAPID-AUTO* (Rigaku, 2001 ▸); cell refinement: *RAPID-AUTO*; data reduction: *RAPID-AUTO*; program(s) used to solve structure: *SIR92* (Altomare *et al.*, 1994 ▸); program(s) used to refine structure: *SHELXL2014* (Sheldrick, 2015 ▸); molecular graphics: *CrystalStructure* (Rigaku, 2010 ▸); software used to prepare material for publication: *CrystalStructure*.

## Supplementary Material

Crystal structure: contains datablock(s) I. DOI: 10.1107/S2056989015022914/su5246sup1.cif

Structure factors: contains datablock(s) I. DOI: 10.1107/S2056989015022914/su5246Isup2.hkl

**Fig. 1.**
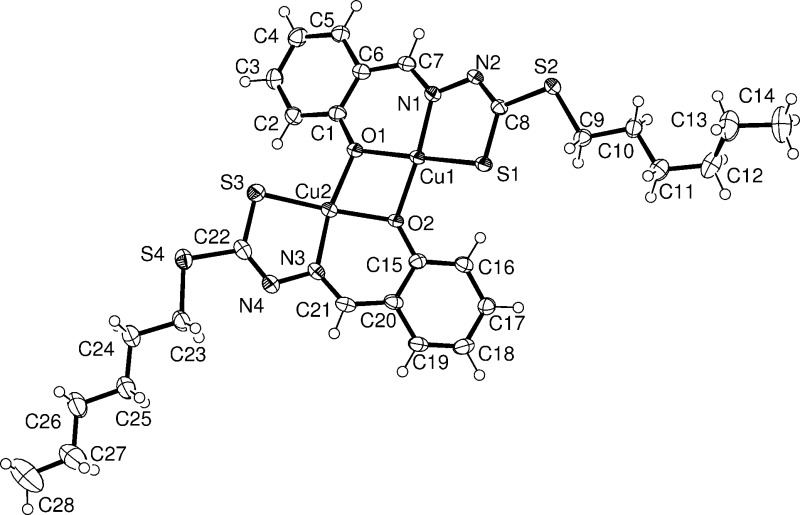
A view of the molecular structure of the title complex, with atom labelling. The displacement ellipsoids are drawn at the 50% probability level.

. DOI: 10.1107/S2056989015022914/su5246fig1.tif
A view of the mol­ecular structure of the title complex, with atom labelling. The displacement ellipsoids are drawn at the 50% probability level.

**Fig. 2.**
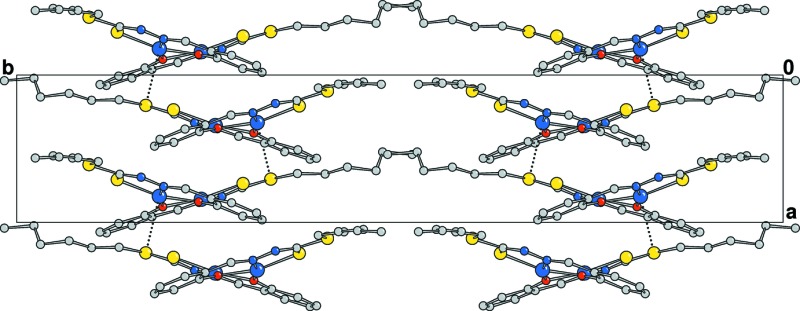
A view along the c axis of the crystal packing of the title complex. Dotted lines indicated the Cu2—S2i distances of 2.9740 (11) Å [symmetry code: (i) x - 1/2, -y + 3/2, z + 1/2], and H atoms have been omitted for clarity.

Click here for additional data file.c i x y z . DOI: 10.1107/S2056989015022914/su5246fig2.tif
A view along the *c* axis of the crystal packing of the title complex. Dotted lines indicated the Cu2—S2^i^ distances of 2.9740 (11) Å [symmetry code: (i) *x* − 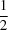, −*y* + 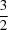, *z* + 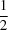], and H atoms have been omitted for clarity.

CCDC reference: 1439650

Additional supporting information:  crystallographic information; 3D view; checkCIF report

## Figures and Tables

**Table 1:** Hydrogen-bond geometry (Å, °)

*D*—H⋯*A*	*D*—H	H⋯*A*	*D*⋯*A*	*D*—H⋯*A*
C19—H19⋯N2^i^	0.95	2.52	3.457 (5)	167

Symmetry code: (i) 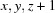.

## supplementary crystallographic information

### S1. Comments

Metal complexes of bidentate Schiff bases of S-methyl or S-benzyl di­thio­carbaza­tes have received considerable attention for their possible bioactivities (Chan *et al.*, 2008; How *et al.*, 2008; Ali *et al.*, 2002; Chew *et al.*, 2004). In square planar metal complexes reported so far di­thio­carbazato ligands coordinate in a bidentate manner through the N,S donors leading to bis­chelated species with a *trans* (Howlader *et al.*, 2015) or *cis* (Begum *et al.*, 2015) configuration. The presence of an oxo­benzyl­idene moiety is expected to induce the ligand to coordinate to the metal through the *N*,*S*,*O* donors. Continuing our studies on S-containing Schiff bases (Howlader *et al.*, 2015; Begum *et al.*, 2015), we report herein on the crystal structure of an unexpected binuclear copper(II) complex of the ligand S-hexyl-β-*N*-(2-hy­droxy­benzyl­idene)di­thio­carbazate.

In the title compound, Fig. 1, the presence of the oxo­benzyl­idene moiety in the Schiff base ligand has induced it to coordinate to the metal through the *N*, *S*, and *O* donor atoms, with formation of five- and six-membered chelate rings. Each oxygen atom bridges to a second copper(II) ion to form a binuclear species having a central Cu_2_O_2_ rhomboid moiety. The bridging angles Cu1—O1—Cu2 and Cu1—O2—Cu2 of 99.23 (12) and 99.69 (12) °, respectively, lead to a metal-metal separation of 2.9923 (6) Å. The Cu—N bond distances of 1.919 (4) and 1.931 (4) Å, and the Cu—S bond distances of 2.2171 (10) and 2.2352 (11) Å, appear slightly shorter by ca. 0.02-0.04 Å than those observed in mononuclear bis­chelated species (Zangrando, Begum, *et al.*, 2015; Zangrando, Islam, *et al.*, 2015; Tarafder *et al.*, 2008). This feature can be ascribed to the double deprotonated ligand in the present case. With exception of the alkyl chains the two chelating ligands have almost coplanar atoms and their mean plane forms a dihedral angle of 34.45 (9)°. It is worth noting that the alkyl chain C23—C28 presents all methyl­ene groups in an *anti* conformation, while the other chain presents a torsion angle C10—C11—C12—C13 of 62.1 (6)°, likely induced by packing requirements. To the best of our knowledge the present complex represents a unique example of a binuclear species with similar tridentate *S*,*N*,*O* ligands derived from S-alkyl­dithio­carbazate, although copper complexes of similar ligands have been reported (Ali, Tan *et al.*, 2012; Ali, Mirza *et al.*, 2012).

In the crystal, the binuclear complexes stack along the *a* axis with all the hexyl chains located side-by-side forming a hydro­phobic region. The complexes are linked via C—H···N hydrogen bonds forming chains along the *c* axis direction (Table 1). Atom Cu2 has the sulfur atom, S2^i^ [code: (i) x - 1/2, -y + 3/2, z+ 1/2], of a symmetry-related complex located approximately in the apical position at 2.9740 (11) Å (Fig. 2). This weak inter­action links the chains to form slabs parallel to the ac plane.

### S2. Synthesis and crystallization

A solution of Cu(CH_3_COO)_2_.H_2_O (0.11 g, 0.5 mmol, 15 ml methanol) was added to a solution of the *S*-hexyl-β-*N*-(2-hy­droxy­benzyl­idene)di­thio­carbazate (1.0 mmol, 10 ml methanol). The resulting mixture was stirred at room temperature for 5 h. A dark reddish brown precipitate was formed, filtered off, washed with methanol and dried in vacuo over anhydrous CaCl_2_. Dark reddish brown single crystals suitable for X-ray diffraction were obtained by slow evaporation from a mixture of di­chloro­methane and aceto­nitrile (3:1); m.p. 443 K.

### S3. Refinement

Crystal data, data collection and structure refinement details are summarized in Table 2. All H atoms were fixed geometrically (C—H = 0.95 - 0.99 Å) and refined as riding with *U*_iso_(H) = 1.2*U*_eq_(C).

### Crystal data

**Table:**

[Cu_2_(C_14_H_18_N_2_OS_2_)_2_]	*F*(000) = 1480
*M**_r_* = 715.93	*D*_x_ = 1.532 Mg m^−^^3^
Monoclinic, *C**c*	Mo *K*α radiation, λ = 0.71075 Å
*a* = 7.2792 (4) Å	Cell parameters from 701 reflections
*b* = 37.7252 (16) Å	θ = 3.2–26.4°
*c* = 11.3443 (5) Å	µ = 1.67 mm^−^^1^
β = 94.701 (2)°	*T* = 173 K
*V* = 3104.8 (3) Å^3^	Platelet, brown
*Z* = 4	0.36 × 0.34 × 0.03 mm

### Data collection

**Table:**

Rigaku R-AXIS RAPID diffractometer	5114 reflections with *I* > 2σ(*I*)
Detector resolution: 10.000 pixels mm^-1^	*R*_int_ = 0.025
ω scans	θ_max_ = 25.4°, θ_min_ = 3.2°
Absorption correction: multi-scan (*ABSCOR*; Higashi, 1995)	*h* = −8→8
*T*_min_ = 0.723, *T*_max_ = 0.951	*k* = −45→45
12702 measured reflections	*l* = −13→13
5278 independent reflections	

### Refinement

**Table:**

Refinement on *F*^2^	Hydrogen site location: inferred from neighbouring sites
Least-squares matrix: full	H-atom parameters constrained
*R*[*F*^2^ > 2σ(*F*^2^)] = 0.027	*w* = 1/[σ^2^(*F*_o_^2^) + (0.0398*P*)^2^] where *P* = (*F*_o_^2^ + 2*F*_c_^2^)/3
*wR*(*F*^2^) = 0.066	(Δ/σ)_max_ = 0.001
*S* = 1.06	Δρ_max_ = 0.60 e Å^−^^3^
5278 reflections	Δρ_min_ = −0.29 e Å^−^^3^
361 parameters	Absolute structure: Flack *x* determined using 2223 quotients [(*I*^+^)-(*I*^-^)]/[(*I*^+^)+(*I*^-^)] (Parsons *et al.*, 2013)
2 restraints	Absolute structure parameter: 0.006 (6)

### Special details

**Table:**

Geometry. All e.s.d.'s (except the e.s.d. in the dihedral angle between two l.s. planes) are estimated using the full covariance matrix. The cell e.s.d.'s are taken into account individually in the estimation of e.s.d.'s in distances, angles and torsion angles; correlations between e.s.d.'s in cell parameters are only used when they are defined by crystal symmetry. An approximate (isotropic) treatment of cell e.s.d.'s is used for estimating e.s.d.'s involving l.s. planes.
Refinement. Refinement was performed using all reflections. The weighted *R*-factor (*wR*) and goodness of fit (*S*) are based on *F*^2^. *R*-factor (gt) are based on *F*. The threshold expression of *F*^2^ > 2.0 σ(*F*^2^) is used only for calculating *R*-factor (gt).

### Fractional atomic coordinates and isotropic or equivalent isotropic displacement parameters (Å^2^)

**Table:**

	*x*	*y*	*z*	*U*_iso_*/*U*_eq_	
Cu1	0.15703 (6)	0.75915 (2)	0.15440 (4)	0.02305 (13)	
Cu2	0.17703 (6)	0.81382 (2)	0.34616 (4)	0.02522 (13)	
S1	0.26421 (15)	0.70459 (3)	0.13607 (8)	0.0267 (2)	
S2	0.29460 (14)	0.66844 (3)	−0.10024 (8)	0.0271 (2)	
S3	0.29144 (15)	0.86872 (3)	0.36366 (9)	0.0318 (2)	
S4	0.39250 (16)	0.90558 (3)	0.58598 (10)	0.0330 (2)	
O1	0.1071 (4)	0.80939 (7)	0.1742 (2)	0.0258 (7)	
O2	0.1407 (4)	0.76259 (7)	0.3249 (2)	0.0257 (7)	
N1	0.1368 (5)	0.76188 (8)	−0.0151 (3)	0.0235 (8)	
N2	0.1776 (5)	0.73220 (9)	−0.0820 (3)	0.0255 (7)	
N3	0.2447 (5)	0.80819 (9)	0.5132 (3)	0.0252 (8)	
N4	0.3081 (5)	0.83759 (9)	0.5811 (3)	0.0282 (7)	
C1	0.0484 (6)	0.83258 (10)	0.0903 (4)	0.0249 (8)	
C2	−0.0068 (6)	0.86690 (10)	0.1209 (4)	0.0298 (9)	
H2	−0.0044	0.8733	0.2020	0.036*	
C3	−0.0638 (6)	0.89129 (10)	0.0367 (4)	0.0328 (9)	
H3	−0.0995	0.9143	0.0602	0.039*	
C4	−0.0704 (6)	0.88291 (11)	−0.0835 (4)	0.0347 (10)	
H4	−0.1093	0.9000	−0.1417	0.042*	
C5	−0.0199 (6)	0.84966 (11)	−0.1157 (4)	0.0296 (9)	
H5	−0.0246	0.8438	−0.1974	0.036*	
C6	0.0393 (6)	0.82360 (11)	−0.0312 (3)	0.0252 (8)	
C7	0.0854 (6)	0.78960 (10)	−0.0771 (3)	0.0252 (9)	
H7	0.0776	0.7871	−0.1607	0.030*	
C8	0.2370 (5)	0.70578 (10)	−0.0176 (3)	0.0221 (8)	
C9	0.3329 (6)	0.63379 (10)	0.0105 (4)	0.0285 (9)	
H9A	0.4534	0.6375	0.0559	0.034*	
H9B	0.2355	0.6349	0.0665	0.034*	
C10	0.3300 (7)	0.59759 (10)	−0.0491 (4)	0.0332 (9)	
H10A	0.4213	0.5971	−0.1091	0.040*	
H10B	0.2065	0.5932	−0.0899	0.040*	
C11	0.3752 (7)	0.56854 (11)	0.0424 (4)	0.0384 (10)	
H11A	0.5040	0.5716	0.0761	0.046*	
H11B	0.2939	0.5713	0.1076	0.046*	
C12	0.3525 (8)	0.53115 (12)	−0.0078 (5)	0.0502 (13)	
H12A	0.3759	0.5140	0.0576	0.060*	
H12B	0.2230	0.5280	−0.0403	0.060*	
C13	0.4783 (9)	0.52245 (13)	−0.1038 (6)	0.0617 (16)	
H13A	0.6074	0.5273	−0.0739	0.074*	
H13B	0.4477	0.5381	−0.1726	0.074*	
C14	0.4620 (10)	0.48402 (14)	−0.1440 (7)	0.075 (2)	
H14A	0.5457	0.4797	−0.2058	0.113*	
H14B	0.4947	0.4683	−0.0766	0.113*	
H14C	0.3349	0.4792	−0.1753	0.113*	
C15	0.1172 (6)	0.73824 (10)	0.4088 (3)	0.0234 (8)	
C16	0.0434 (6)	0.70501 (11)	0.3792 (4)	0.0266 (8)	
H16	0.0100	0.6996	0.2985	0.032*	
C17	0.0179 (6)	0.67982 (11)	0.4649 (3)	0.0283 (9)	
H17	−0.0324	0.6573	0.4423	0.034*	
C18	0.0653 (7)	0.68710 (11)	0.5843 (4)	0.0333 (10)	
H18	0.0492	0.6696	0.6430	0.040*	
C19	0.1349 (6)	0.71952 (10)	0.6152 (3)	0.0292 (9)	
H19	0.1661	0.7246	0.6964	0.035*	
C20	0.1624 (6)	0.74617 (10)	0.5296 (4)	0.0266 (8)	
C21	0.2290 (6)	0.77955 (11)	0.5743 (3)	0.0272 (9)	
H21	0.2651	0.7809	0.6566	0.033*	
C22	0.3282 (6)	0.86546 (11)	0.5167 (4)	0.0282 (9)	
C23	0.4128 (7)	0.89347 (12)	0.7416 (4)	0.0357 (10)	
H23A	0.3009	0.8806	0.7615	0.043*	
H23B	0.5205	0.8777	0.7587	0.043*	
C24	0.4365 (7)	0.92718 (12)	0.8150 (4)	0.0399 (11)	
H24A	0.5535	0.9388	0.7985	0.048*	
H24B	0.3345	0.9437	0.7913	0.048*	
C25	0.4385 (7)	0.91990 (12)	0.9463 (4)	0.0394 (11)	
H25A	0.3241	0.9071	0.9614	0.047*	
H25B	0.5437	0.9041	0.9698	0.047*	
C26	0.4533 (9)	0.95249 (13)	1.0232 (5)	0.0572 (15)	
H26A	0.3523	0.9690	0.9970	0.069*	
H26B	0.5714	0.9646	1.0120	0.069*	
C27	0.4440 (12)	0.94475 (19)	1.1539 (5)	0.076 (2)	
H27A	0.3329	0.9302	1.1634	0.091*	
H27B	0.5527	0.9303	1.1814	0.091*	
C28	0.4381 (15)	0.9760 (2)	1.2308 (7)	0.112 (3)	
H28A	0.4322	0.9683	1.3130	0.168*	
H28B	0.5492	0.9903	1.2245	0.168*	
H28C	0.3288	0.9902	1.2064	0.168*	

### Atomic displacement parameters (Å^2^)

**Table:**

	*U*^11^	*U*^22^	*U*^33^	*U*^12^	*U*^13^	*U*^23^
Cu1	0.0294 (3)	0.0239 (2)	0.0163 (2)	0.00168 (19)	0.00456 (18)	−0.00053 (18)
Cu2	0.0311 (3)	0.0258 (2)	0.0191 (2)	−0.0010 (2)	0.00362 (19)	−0.00153 (18)
S1	0.0357 (6)	0.0258 (5)	0.0190 (4)	0.0041 (4)	0.0043 (4)	−0.0001 (4)
S2	0.0330 (5)	0.0255 (5)	0.0233 (5)	0.0004 (4)	0.0064 (4)	−0.0035 (4)
S3	0.0355 (6)	0.0315 (5)	0.0288 (5)	−0.0062 (4)	0.0043 (4)	0.0002 (4)
S4	0.0333 (6)	0.0303 (5)	0.0352 (5)	−0.0036 (4)	0.0025 (4)	−0.0060 (4)
O1	0.0368 (18)	0.0231 (13)	0.0182 (14)	0.0014 (11)	0.0060 (13)	−0.0001 (10)
O2	0.0404 (19)	0.0221 (13)	0.0152 (13)	0.0010 (12)	0.0062 (13)	0.0000 (10)
N1	0.0246 (18)	0.0249 (17)	0.0211 (17)	0.0009 (14)	0.0033 (14)	−0.0018 (13)
N2	0.0339 (19)	0.0258 (17)	0.0175 (16)	0.0003 (15)	0.0058 (14)	−0.0034 (13)
N3	0.0249 (19)	0.0290 (17)	0.0217 (17)	0.0006 (14)	0.0025 (14)	−0.0062 (14)
N4	0.0288 (18)	0.0293 (17)	0.0262 (17)	0.0000 (15)	0.0004 (15)	−0.0040 (14)
C1	0.022 (2)	0.030 (2)	0.0228 (18)	−0.0012 (16)	0.0031 (15)	0.0006 (16)
C2	0.032 (2)	0.030 (2)	0.029 (2)	0.0007 (17)	0.0068 (18)	−0.0032 (16)
C3	0.035 (2)	0.026 (2)	0.038 (2)	0.0032 (18)	0.0027 (19)	−0.0031 (18)
C4	0.037 (2)	0.031 (2)	0.035 (2)	0.0037 (19)	−0.0043 (19)	0.0069 (18)
C5	0.034 (2)	0.030 (2)	0.0239 (19)	−0.0007 (17)	−0.0023 (17)	0.0022 (17)
C6	0.023 (2)	0.031 (2)	0.0218 (19)	−0.0015 (17)	0.0021 (16)	0.0003 (16)
C7	0.024 (2)	0.032 (2)	0.0196 (18)	−0.0026 (17)	0.0035 (16)	−0.0001 (16)
C8	0.0188 (19)	0.0265 (19)	0.0219 (19)	−0.0012 (15)	0.0067 (15)	−0.0016 (15)
C9	0.030 (2)	0.027 (2)	0.030 (2)	−0.0011 (17)	0.0087 (17)	0.0014 (17)
C10	0.038 (2)	0.024 (2)	0.039 (2)	−0.0020 (17)	0.0107 (19)	−0.0021 (17)
C11	0.038 (3)	0.031 (2)	0.047 (3)	0.0040 (19)	0.004 (2)	0.005 (2)
C12	0.048 (3)	0.030 (2)	0.072 (3)	−0.001 (2)	0.006 (3)	0.010 (2)
C13	0.062 (4)	0.038 (3)	0.088 (4)	0.001 (3)	0.022 (3)	−0.010 (3)
C14	0.077 (5)	0.040 (3)	0.110 (6)	0.004 (3)	0.006 (4)	−0.022 (3)
C15	0.023 (2)	0.025 (2)	0.0232 (19)	0.0055 (15)	0.0076 (16)	0.0023 (15)
C16	0.028 (2)	0.031 (2)	0.0214 (18)	0.0040 (17)	0.0047 (16)	−0.0016 (16)
C17	0.030 (2)	0.027 (2)	0.028 (2)	0.0006 (17)	0.0053 (17)	0.0007 (16)
C18	0.039 (3)	0.034 (2)	0.028 (2)	0.0077 (19)	0.0099 (19)	0.0101 (17)
C19	0.034 (2)	0.035 (2)	0.0196 (18)	0.0064 (18)	0.0042 (16)	0.0023 (16)
C20	0.028 (2)	0.032 (2)	0.0205 (19)	0.0081 (17)	0.0069 (16)	−0.0001 (16)
C21	0.026 (2)	0.038 (2)	0.0183 (18)	0.0055 (18)	0.0022 (16)	0.0006 (17)
C22	0.020 (2)	0.034 (2)	0.031 (2)	−0.0013 (16)	0.0036 (17)	−0.0095 (18)
C23	0.039 (3)	0.034 (2)	0.033 (2)	−0.006 (2)	0.002 (2)	−0.0093 (19)
C24	0.047 (3)	0.034 (2)	0.038 (2)	−0.002 (2)	0.004 (2)	−0.010 (2)
C25	0.040 (3)	0.037 (2)	0.041 (2)	−0.003 (2)	0.008 (2)	−0.011 (2)
C26	0.083 (4)	0.040 (3)	0.048 (3)	0.006 (3)	−0.001 (3)	−0.015 (2)
C27	0.103 (6)	0.078 (4)	0.047 (3)	0.005 (4)	0.008 (4)	−0.016 (3)
C28	0.134 (8)	0.128 (7)	0.074 (5)	0.005 (6)	0.017 (5)	−0.048 (5)

### Geometric parameters (Å, º)

**Table:**

Cu1—N1	1.919 (4)	C11—H11A	0.9900
Cu1—O1	1.946 (3)	C11—H11B	0.9900
Cu1—O2	1.952 (3)	C12—C13	1.515 (8)
Cu1—S1	2.2171 (10)	C12—H12A	0.9900
Cu1—Cu2	2.9923 (6)	C12—H12B	0.9900
Cu2—N3	1.931 (4)	C13—C14	1.522 (7)
Cu2—O2	1.963 (2)	C13—H13A	0.9900
Cu2—O1	1.982 (3)	C13—H13B	0.9900
Cu2—S3	2.2352 (11)	C14—H14A	0.9800
S1—C8	1.739 (4)	C14—H14B	0.9800
S2—C8	1.762 (4)	C14—H14C	0.9800
S2—C9	1.819 (4)	C15—C16	1.394 (6)
S3—C22	1.739 (4)	C15—C20	1.415 (6)
S4—C22	1.751 (4)	C16—C17	1.383 (6)
S4—C23	1.817 (4)	C16—H16	0.9500
O1—C1	1.337 (5)	C17—C18	1.398 (6)
O2—C15	1.343 (5)	C17—H17	0.9500
N1—C7	1.299 (5)	C18—C19	1.359 (6)
N1—N2	1.399 (5)	C18—H18	0.9500
N2—C8	1.289 (5)	C19—C20	1.423 (6)
N3—C21	1.293 (5)	C19—H19	0.9500
N3—N4	1.406 (5)	C20—C21	1.428 (6)
N4—C22	1.295 (5)	C21—H21	0.9500
C1—C2	1.407 (6)	C23—C24	1.522 (6)
C1—C6	1.416 (5)	C23—H23A	0.9900
C2—C3	1.366 (6)	C23—H23B	0.9900
C2—H2	0.9500	C24—C25	1.513 (6)
C3—C4	1.396 (6)	C24—H24A	0.9900
C3—H3	0.9500	C24—H24B	0.9900
C4—C5	1.366 (6)	C25—C26	1.507 (6)
C4—H4	0.9500	C25—H25A	0.9900
C5—C6	1.415 (6)	C25—H25B	0.9900
C5—H5	0.9500	C26—C27	1.517 (8)
C6—C7	1.434 (6)	C26—H26A	0.9900
C7—H7	0.9500	C26—H26B	0.9900
C9—C10	1.524 (5)	C27—C28	1.468 (9)
C9—H9A	0.9900	C27—H27A	0.9900
C9—H9B	0.9900	C27—H27B	0.9900
C10—C11	1.527 (6)	C28—H28A	0.9800
C10—H10A	0.9900	C28—H28B	0.9800
C10—H10B	0.9900	C28—H28C	0.9800
C11—C12	1.525 (6)		
			
N1—Cu1—O1	93.65 (12)	H11A—C11—H11B	107.7
N1—Cu1—O2	169.54 (14)	C13—C12—C11	114.5 (4)
O1—Cu1—O2	78.10 (11)	C13—C12—H12A	108.6
N1—Cu1—S1	87.40 (10)	C11—C12—H12A	108.6
O1—Cu1—S1	170.18 (10)	C13—C12—H12B	108.6
O2—Cu1—S1	101.83 (8)	C11—C12—H12B	108.6
N1—Cu1—Cu2	133.36 (9)	H12A—C12—H12B	107.6
O1—Cu1—Cu2	40.83 (8)	C12—C13—C14	112.6 (5)
O2—Cu1—Cu2	40.28 (7)	C12—C13—H13A	109.1
S1—Cu1—Cu2	135.36 (3)	C14—C13—H13A	109.1
N3—Cu2—O2	91.89 (13)	C12—C13—H13B	109.1
N3—Cu2—O1	168.85 (12)	C14—C13—H13B	109.1
O2—Cu2—O1	77.02 (11)	H13A—C13—H13B	107.8
N3—Cu2—S3	87.22 (11)	C13—C14—H14A	109.5
O2—Cu2—S3	165.65 (10)	C13—C14—H14B	109.5
O1—Cu2—S3	103.25 (9)	H14A—C14—H14B	109.5
N3—Cu2—Cu1	129.13 (10)	C13—C14—H14C	109.5
O2—Cu2—Cu1	40.03 (8)	H14A—C14—H14C	109.5
O1—Cu2—Cu1	39.94 (8)	H14B—C14—H14C	109.5
S3—Cu2—Cu1	134.33 (3)	O2—C15—C16	120.9 (3)
C8—S1—Cu1	93.33 (13)	O2—C15—C20	120.6 (3)
C8—S2—C9	103.67 (19)	C16—C15—C20	118.5 (4)
C22—S3—Cu2	92.80 (14)	C17—C16—C15	121.3 (4)
C22—S4—C23	102.5 (2)	C17—C16—H16	119.3
C1—O1—Cu1	127.4 (3)	C15—C16—H16	119.3
C1—O1—Cu2	133.4 (2)	C16—C17—C18	120.6 (4)
Cu1—O1—Cu2	99.23 (12)	C16—C17—H17	119.7
C15—O2—Cu1	132.7 (2)	C18—C17—H17	119.7
C15—O2—Cu2	127.6 (2)	C19—C18—C17	119.0 (4)
Cu1—O2—Cu2	99.69 (12)	C19—C18—H18	120.5
C7—N1—N2	114.5 (3)	C17—C18—H18	120.5
C7—N1—Cu1	125.6 (3)	C18—C19—C20	122.0 (4)
N2—N1—Cu1	119.9 (2)	C18—C19—H19	119.0
C8—N2—N1	112.8 (3)	C20—C19—H19	119.0
C21—N3—N4	113.9 (3)	C15—C20—C19	118.5 (4)
C21—N3—Cu2	126.2 (3)	C15—C20—C21	125.1 (4)
N4—N3—Cu2	119.8 (3)	C19—C20—C21	116.3 (4)
C22—N4—N3	112.2 (3)	N3—C21—C20	126.1 (4)
O1—C1—C2	120.5 (4)	N3—C21—H21	117.0
O1—C1—C6	121.6 (4)	C20—C21—H21	117.0
C2—C1—C6	117.9 (4)	N4—C22—S3	127.2 (3)
C3—C2—C1	121.6 (4)	N4—C22—S4	119.1 (3)
C3—C2—H2	119.2	S3—C22—S4	113.6 (2)
C1—C2—H2	119.2	C24—C23—S4	108.5 (3)
C2—C3—C4	121.0 (4)	C24—C23—H23A	110.0
C2—C3—H3	119.5	S4—C23—H23A	110.0
C4—C3—H3	119.5	C24—C23—H23B	110.0
C5—C4—C3	118.8 (4)	S4—C23—H23B	110.0
C5—C4—H4	120.6	H23A—C23—H23B	108.4
C3—C4—H4	120.6	C25—C24—C23	112.2 (4)
C4—C5—C6	122.0 (4)	C25—C24—H24A	109.2
C4—C5—H5	119.0	C23—C24—H24A	109.2
C6—C5—H5	119.0	C25—C24—H24B	109.2
C5—C6—C1	118.7 (4)	C23—C24—H24B	109.2
C5—C6—C7	116.3 (4)	H24A—C24—H24B	107.9
C1—C6—C7	125.0 (4)	C26—C25—C24	114.6 (4)
N1—C7—C6	126.1 (4)	C26—C25—H25A	108.6
N1—C7—H7	117.0	C24—C25—H25A	108.6
C6—C7—H7	117.0	C26—C25—H25B	108.6
N2—C8—S1	126.4 (3)	C24—C25—H25B	108.6
N2—C8—S2	113.6 (3)	H25A—C25—H25B	107.6
S1—C8—S2	120.0 (2)	C25—C26—C27	113.7 (5)
C10—C9—S2	110.0 (3)	C25—C26—H26A	108.8
C10—C9—H9A	109.7	C27—C26—H26A	108.8
S2—C9—H9A	109.7	C25—C26—H26B	108.8
C10—C9—H9B	109.7	C27—C26—H26B	108.8
S2—C9—H9B	109.7	H26A—C26—H26B	107.7
H9A—C9—H9B	108.2	C28—C27—C26	115.6 (6)
C9—C10—C11	110.3 (4)	C28—C27—H27A	108.4
C9—C10—H10A	109.6	C26—C27—H27A	108.4
C11—C10—H10A	109.6	C28—C27—H27B	108.4
C9—C10—H10B	109.6	C26—C27—H27B	108.4
C11—C10—H10B	109.6	H27A—C27—H27B	107.4
H10A—C10—H10B	108.1	C27—C28—H28A	109.5
C12—C11—C10	113.5 (4)	C27—C28—H28B	109.5
C12—C11—H11A	108.9	H28A—C28—H28B	109.5
C10—C11—H11A	108.9	C27—C28—H28C	109.5
C12—C11—H11B	108.9	H28A—C28—H28C	109.5
C10—C11—H11B	108.9	H28B—C28—H28C	109.5

### Hydrogen-bond geometry (Å, º)

**Table:**

*D*—H···*A*	*D*—H	H···*A*	*D*···*A*	*D*—H···*A*
C19—H19···N2^i^	0.95	2.52	3.457 (5)	167

Symmetry code: (i) *x*, *y*, *z*+1.
