# Supplementary figures and images for: Crystal structure of bis­[μ-S-hexyl 3-(2-oxido­benzyl­idene)di­thio­carbazato-κ4 O,N 3,S:O]dicopper(II)
Source: Acta Crystallogr E Crystallogr Commun. 2015 Dec 9;71(Pt 12):m249–50. doi: 10.1107/S2056989015022914 (PMC4719858; doi:10.1107/S2056989015022914)

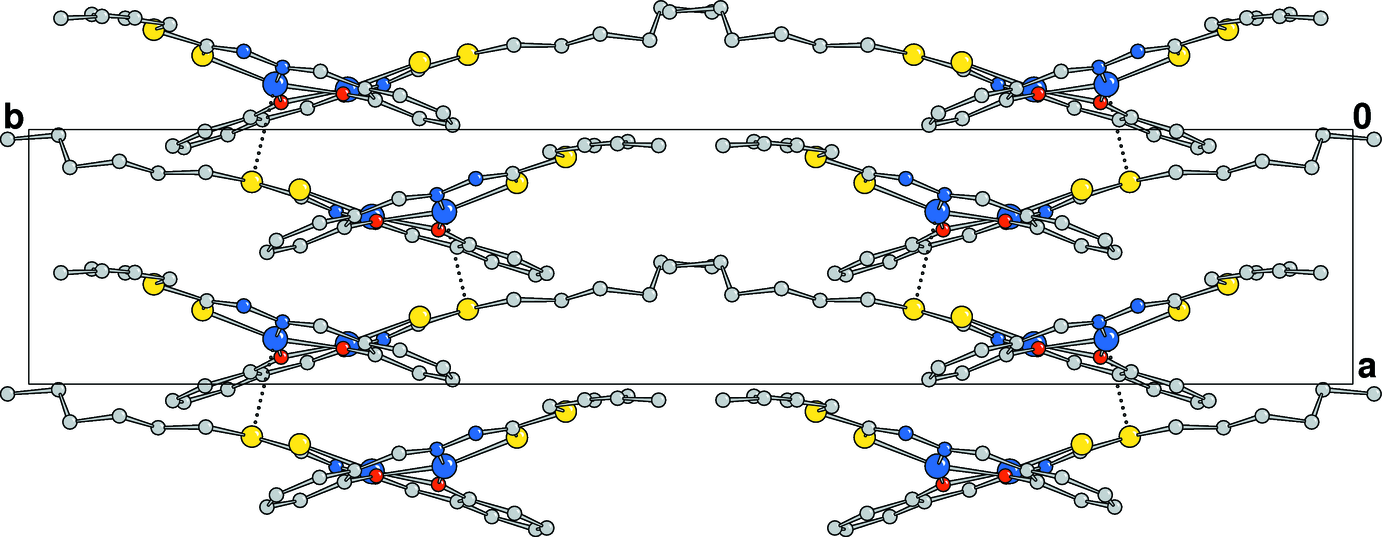

Supplement: Supplementary file 4 [file e-71-0m249-fig2.tif]
